# Our Contemporaries

**Published:** 1916-12

**Authors:** 


### OUR CONTEMPORARIES

---

### "A Specious But Vicious Principle."

"Dr. J. Bayard Clark, of New York, published an interesting article in the July issue of the New York State Journal of Medicine on the self-supporting hospital. He showed just how such an institution could be made to meet all its expenses and also pay salaries to its medical and surgical staffs, whose offices would be in the building.

"This is surely the last word among all the schemes to prevent the general practitioner from being self-supporting. We must have self-supporting hospitals, and self-supporting staffs, but it remains for someone to show how the general practitioner may become self-supporting. Were such hospitals as Dr. Clark proposes to become numerous no practitioner on the outside would stand much chance of survival.

"Sometimes the psychology of such schemes seems to stand revealed as a desire to draw coteries together which shall be exempt from competition. Do not such schemes mean professional decadence, with the weak unconsciously storing honey for themselves while they poison the supplies of the rank and file?"—Medical Times, Nev. Is the Times correct in its assumption of the cloven hoof?

---

The Texas Medical Journal (The Red Back) will celebrate its 25th anniversary by changing its name to Medical Insurance and Health Conservation and will enter the national field of journalism. The proprietor and editor is Mrs. F. E. Daniels.

---

The Medico-Legal Journal, Sept., holds that WHITE SLAVERY is about as prevalent as ever and no more so; that the street walker or professional prostitute who supports her lover and protector, is not a white slave, and that the male pimp is on about the same level as the married man who allows his wife to support him by taking in washing; that the published stories of white slavery are mainly furnished by prostitutes who need no sympathy; that the real white slave rarely exists and is not turned loose on the streets but is kept half drunk and drugged in a house; that there is hardly any doubt but that some girls are born to be prostitutes; that the sex instinct is stronger in children than is realized and that parental control is necessary, aided by a curfew law.

---

Dr. E. P. Anshutz, Editor of the Homoeopathic Recorder in quoting from this journal calls us brother and speaks of the publication as ''that fine old Journal.'' We reciprocate the fraternal greeting. There is more real ethics in a cordiality of this kind than in the whole of the old code that emphasized the differences between methods of practice and that ignored the essentials of professional life and aspirations. Be it remembered, too, that the Recorder is homoeopathic in belief as well as in name and that the respective editorial brethren disagree as to medical theory and practice about as thoroughly as could be imagined. Friendly disagreement coupled with mutual respect is a strong factor in ultimate agreement as to fact.

